# Regulatory Harmonization Needs for Farm-to-Fork Bacteriophage Applications in South American Food Systems

**DOI:** 10.3390/foods15112031

**Published:** 2026-06-05

**Authors:** Boris Parra, Roberto Bastías, Gastón Higuera, Inés Marlene Rosales, Lorena Galarce, Víctor Rivera, Kasim Allel, Marta Fonseca-Martins, Martha J. Vives F, Judy Natalia Jiménez, Natalia Echeverría, Carolina Marambio, Daniel Aguayo, Gerardo González-Rocha, Aura Villamil, Marcela Fresno, Dácil Rivera

**Affiliations:** 1 Núcleo de Microbiología Traslacional Para la Vigilancia e Innovación en Sistemas Sanitarios, Ambientales y Productivos MICRA, Facultad de Medicina Veterinaria y Agronomía, Universidad de las Américas, Av. Manuel Montt 948, Santiago 7500908, Chile; bparra@udla.cl (B.P.); avillamil@udla.cl (A.V.); 2 Instituto de Biología, Pontificia Universidad Católica de Valparaíso, Av. Universidad #330, Valparaíso 2520000, Chile; roberto.bastias@pucv.cl; 3 Plant Biology and Agro-Food Systems Innovation Laboratory, Institute of Nutrition and Food Technology, University of Chile, Av. El Líbano 5524, Macul, Santiago 7830490, Chile; gastonhiguera@inta.uchile.cl; 4 Departamento de Ciencias Vegetales, Facultad de Agronomía y Sistemas Naturales, Pontificia Universidad Católica de Chile, Av. Vicuña Mackenna 4860, Macul, Santiago 7820436, Chile; irosalesv@uc.cl; 5 Asesora en Asuntos Internacionales y Regulatorios, Agencia Chilena para la Calidad e Inocuidad Alimentaria, Calle Nueva York 17, piso 4, Santiago 8320000, Chile; lorena.galarce@achipia.gob.cl; 6 Área de Asuntos Internacionales y Regulatorios, Agencia Chilena Para la Calidad e Inocuidad Alimentaria, Calle Nueva York 17, piso 4, Santiago 8320000, Chile; victor.rivera@achipia.gob.cl; 7 Department of Infectious Diseases, Pontificia Universidad Católica de Chile, Marcoleta 367, Santiago 8320165, Chile; kasim.allel@gmail.com; 8 Molecular Genetics Laboratory, Embrapa Dairy Cattle, Eugênio do Nascimento Avenue, 610—Aeroporto, Juiz de Fora 36038-330, MG, Brazil; marta.martins@embrapa.br; 9 Department of Biological Sciences, Universidad de Los Andes, Bogotá 111711, Colombia; mvives@uniandes.edu.co; 10 Research Group in Basic and Applied Microbiology (MICROBA), School of Microbiology, University of Antioquia, Medellín 050010, Colombia; jnatalia.jimenez@udea.edu.co; 11 Laboratory of Molecular Virology, Centro de Investigaciones Nucleares, Facultad de Ciencias, Universidad de la República Montevideo, Montevideo 11400, Uruguay; necheverria@fcien.edu.uy; 12 Laboratory of Experimental Evolution of Viruses, Institut Pasteur Montevideo, Mataojo 2020, Montevideo 11400, Uruguay; 13 Subdepartamento Registro y Control Medicamentos Veterinarios, División Protección Pecuaria, Servicio Agrícola y Ganadero, Avenida Bulnes N° 140, Santiago Centro 8330508, Chile; carolina.marambio@sag.gob.cl; 14 ITISB—Instituto de Tecnología Para la Innovación en Salud y Bienestar, Facultad de Ingeniería. Universidad Andrés Bello, Calle 1 Oriente #1180, 5to piso, Viña del Mar 2520000, Chile; daniel.aguayo@unab.cl; 15 Laboratorio de Investigación en Agentes Antibacterianos (LIAA), Departamento de Microbiología, Facultad de Ciencias Biológicas, Universidad de Concepción, Barrio Universitario s/n, Concepción 4070386, Chile; ggonzal@udec.cl; 16 Centro de Modelamiento y Perspectiva One Health, Facultad de Medicina Veterinaria y Agronomía, Universidad de Las Américas, Av. Manuel Montt 948, Santiago 7500908, Chile; mfresno@udla.cl

**Keywords:** bacteriophages, phage control, farm-to-fork, antimicrobial resistance, Regulatory Harmonization, Codex Alimentarius, food systems

## Abstract

Bacteriophage-based products are gaining attention as effective tools to reduce harmful germs in food and combat antimicrobial resistance throughout the food production process. However, in South America, their use is still limited because of complicated regulations and inconsistent evidence requirements. This review aims to (i) explore the current scientific and technological landscape of using bacteriophages in South American food systems, (ii) identify main regulatory challenges that impact their classification, approval, and use, and (iii) highlight the need for consistent international guidelines, especially from Codex Alimentarius, to help safely and effectively incorporate phage-based products in food. Research on phage-based products is growing, but it is not consistent across different regions. There are more patents and advancements in biotechnology, but they are limited to certain areas. Although progress is being made, the regulatory frameworks are still unclear, especially when it comes to how these products are classified, labeled, and monitored for safety. To address these gaps, risk-based guidelines are needed. These should define product categories and claims, set safety standards, and include rules for tracking products and monitoring them after they hit the market. Creating a new Codex Alimentarius project on phage-based products could help establish global guidelines that promote safe use, reduce uncertainty in regulations, and improve trade in food markets around the world.

## 1. Introduction

Food is a major way that harmful bacteria spread, causing serious diseases in both people and animals. Some key bacteria that cause foodborne illnesses include *Salmonella*, *Campylobacter*, *Escherichia coli*, and *Listeria monocytogenes* [1]. Foodborne diseases are a significant public health issue worldwide. They lead to economic losses, higher healthcare costs, negative effects on child development, lower productivity, and disruptions to international trade [2]. Each year, around 600 million people get sick from these diseases, resulting in about 420,000 deaths [3]. The impact of foodborne diseases is greater in low- and middle-income countries, where weak food safety systems lead to 1200 disability-adjusted life years (DALYs) lost for every 100,000 people. In high-income countries, this number is only 30 to 50 DALYs [1].

Research shows that food can spread harmful bacteria and also contribute to the spread of antimicrobial resistance (AMR) [4]. In 2021, AMR was linked to about 4.71 million deaths worldwide. By 2050, it could directly cause around 1.91 million deaths and add to a total of 8.22 million deaths globally [5]. In South America, AMR has become a serious problem, especially with a significant rise in carbapenem resistance among Gram-negative bacteria (from 0.3% in 2002 to 21% in 2016) [6]. Many countries in the region report prevalence rates between 20% and 50% [7]. The mortality rate related to AMR is among the highest in this area, with about 322,000 deaths in 2021. This number is expected to rise to 650,000 deaths each year by 2050 [8].

Antimicrobial resistance (AMR) is becoming a serious economic issue. The World Bank estimates that if it is not controlled, AMR could greatly reduce global GDP by 2050 and push millions of people into poverty, especially in low- and middle-income areas [9]. This problem is not just about public health; it also poses a risk to food production and safety. As a result, there are growing regulatory pressures on food systems worldwide.

Antimicrobial-resistant bacteria in food is a complicated issue, mainly caused by the excessive and incorrect use of antibiotics in farming and animal production. These practices lead to the growth of resistant bacteria in animals raised for food. These bacteria can later get into the food we eat, increasing the chance of cross-contamination in animal products, helping also the spread resistant infections in communities [10].

In South America, the food production system is a major source of antibiotic-resistant pathogens [4]. This includes certain strains of *E. coli* in meat and dairy products that produce extended-spectrum β-lactamase [4,11], and *E. coli* in poultry that is resistant to quinolones. There are also non-typhoidal *Salmonella* types in poultry and eggs that are resistant to cephalosporins, quinolones, and tetracyclines [4,12]. Additionally, antibiotic-resistant *Campylobacter* strains have been found in poultry production [4,13]. Methicillin-resistant *Staphylococcus aureus* (MRSA) is a big worry among Gram-positive bacteria, especially due to its strong link to livestock [4,14].

These findings emphasize the need for effective strategies to prevent and control problems throughout the entire food production process [1,15]. So, a farm-to-fork approach is key to making sure the food that reaches consumers is safe and does not carry antibiotic-resistant bacteria.

As antibiotic resistance increases, policies to reduce antibiotic use and stricter food safety rules are pushing the development of specific biocontrol methods. These methods can lower bacterial levels without the side effects that come with broad-spectrum antibiotics [3]. Together, these factors show that we need innovative, targeted, and scalable solutions that can reduce bacteria while keeping the microbial balance intact and minimizing environmental harm [3].

Bacteriophage (phage)-based methods are a promising alternative to traditional antibiotics. Lytic phages can be used throughout the food production process, from the farm to fork [16,17,18,19,20]. They can help control bacteria on the farm, manage waste like water and manure, improve food processing environments, and even be applied directly to food, following certain regulations [17,20].

Phage-based products [17,20], including phages and endolysins [21], can be used at various stages of food production, including treating the final product in its packaging. These applications can be categorized into two main groups: pre-harvest, which involves crop cultivation and animal production, and post-harvest, which refers to processes within the food supply chain [17].

Depending on their purpose, phage applications can be classified as biocontrol, biopreservation, or biosanitation methods and can be applied either before or after harvesting [20]. Given their versatility and the growing concern about antibiotic-resistant bacteria in the food supply, phage interventions fit well within a One Health approach. This means they can help address health issues across connected environments and shared systems [22].

South America constitutes one of the world’s leading food-exporting regions. Therefore, to keep food safe from the farm to the table, it is crucial to manage food safety effectively. In this context, phages are useful because they can reduce harmful pathogens and limit the use of antibiotics without disrupting production and trade and helping to strengthen the economies that rely on exports [23,24]. However, trade regulations can disrupt this process. Therefore, it is important to have solutions that can be easily adopted in the market. Clear global guidelines are necessary to avoid inconsistent regulations in different countries. This helps ensure that major importing markets have straightforward rules, which reduces uncertainty and avoids obstacles for South American exporters [25,26].

At the global level, the main markets for food exports from this region are the United States and the European Union. It is important to understand how phage-based products are regulated in these areas. These products are not regulated by specific phage laws but are instead classified under existing regulations, which vary by region.

In the United States, the FDA can approve phage-based bioproducts, including through a special process known as Generally Recognized as Safe (GRAS) [27,28]. Canada, Australia, and New Zealand have similar pathways for these products. However, in the European Union, phage-based bioproducts are evaluated individually by the European Food Safety Authority (EFSA), and there is no general approval category like the Qualified Presumption of Safety (QPS) [29,30,31]. In this context new initiatives, especially within the European Medicines Agency, highlight the need for flexible regulations that can adapt to the unique nature of phage-based products (Table S1) [32].

On the other hand, South America still lacks comprehensive regulatory frameworks governing the use of phages in food systems, and only limited pathways to commercialization have been established. Although some countries have issued specific approvals or conducted pilot applications, no harmonized regional guideline currently exists. At the same time, several South American countries are beginning to explore phage biocontrol more actively [33]. More broadly, several bacteriophage-based products are already being marketed, particularly as veterinary or zootechnical solutions. However, regulatory oversight is generally implemented through existing national frameworks, such as feed additives, veterinary products, or biopesticides, rather than through harmonized, phage-specific food legislation. As a result, authorizations remain country-specific and heterogeneous, and publicly accessible regulatory identifiers are not consistently available [16]. This fragmented regulatory context creates uncertainty for developers, limits investment and technology transfer, and hinders the scalability of phage-based solutions across production systems. Moreover, in the absence of clear health authorization pathways, confidence among food producers and consumers may remain limited, further complicating the integration of these technologies into export-oriented supply chains, particularly in markets with more stringent regulatory requirements. For instance, Chile has received specific approval to sell phages (e.g., INSPEKTOR^®^) [34], while Brazil has approved a phage product for use in animal feed, similar to how the EU’s EFSA operates (e.g., Bafasal^®^) [35]. Argentina has set up some regulatory groundwork for phage approval but has not yet approved any products. Pilot studies have been conducted in Colombia, Peru, Ecuador, Uruguay, Paraguay, and Venezuela for certain uses, such as aquaculture, but there are no national approvals at this time. These points highlight the need to address the challenges and the importance of establishing a broader approval system for phage production and use in South America to promote the commercialization of phage-based products across the region.

This review looks at how bacteriophages are used in food systems in South America. It highlights the main regulatory challenges for their classification and approval. The review also emphasizes the need for consistent international guidelines, especially through Codex Alimentarius, to support their safe and widespread use from farms to consumers [36].

## 2. Regulatory Difficulty in Classifying Phage-Based Bioproducts and Labeling

Two main aspects determine the classification of phage-based products. First, classification is based on their intended use. This can include processing aids, food additives or preservatives, decontamination agents for animal foods, or phage products for surfaces in food facilities. Second, classification also depends on how health authorities approve their use. This includes pathways such as GRAS [27,28], processing aids, or authorization upon request. The approval process varies; for example, it is generally more flexible in South America compared to the more standardized EFSA system [29,30,31]. This lack of consistency can be a challenge when trying to get new products approved by regulatory authorities. Accordingly, the key definitions used to classify phage-based bioproducts across the farm-to-fork continuum are summarized below.

### 2.1. Processing Aids

**Figure 1:**
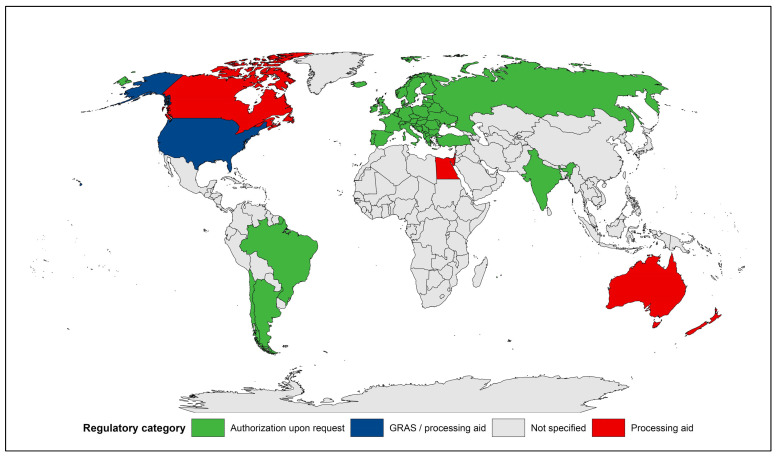
Global overview of regulatory pathways for bacteriophage-based products. Countries and regions are colored according to the main regulatory route through which bacteriophage-based applications may currently be assessed. Blue indicates jurisdictions where phages may be evaluated through pathways such as GRAS and/or processing-aid-related approaches, as illustrated by the United States. Red indicates jurisdictions with recognized processing aid frameworks, including Canada, Australia, Egypt and New Zealand. Green indicates jurisdictions where authorization may proceed upon request or through case-by-case assessment under existing regulatory mechanisms, including the European Union, India, and South America, as represented in this schematic. This classification is intended to summarize broad regulatory tendencies and does not imply the existence of a harmonized or phage-specific approval pathway across all countries within each region.

Processing aids are substances utilized during food processing for technological purposes and lack nutritional value. Consequently, phages may be present in the final product as unavoidable residues, provided they are harmless to health, do not affect the technological properties of the product (such as color, odor, or taste), and are inactive. However, demonstrating their absolute absence can be challenging, as phages can remain within bacterial cells. This classification is recognized in some countries outside the EU and does not require labeling [18] (Figure 1; Table S1).

### 2.2. Food Additives/Preservatives

Food additives are classified into functional categories. Bacteriophages fall under the category of preservatives, which are substances that extend shelf life by protecting food from microorganisms or inhibiting harmful pathogens. However, due to their high specificity, phage cocktails cannot be directly compared to conventional preservatives [20].

In the European Union, a key requirement is that each individual phage or phage cocktail must undergo separate authorization. This process includes providing evidence of specificity, efficacy, duration of action, and clearly defined applications based on host range. Unlike processing aids, food additives must be approved and are subject to mandatory labeling. Therefore, phages would be considered components of the final food product, as their removal is not anticipated [18].

In the United States, phage products may be authorized through a Food Additive Petition (FAP), with GRAS submissions occurring prior to classification under food additive regulations. In certain cases, regulatory adjustments may allow for the updating of phage cocktails without the need to submit a new FAP [27,28].

### 2.3. Decontamination Agents (Biocides) for Foods of Animal Origin

Agents used to remove microbial surface contamination from foods of animal origin are subject to specific rules under Regulation (EC) No 853/2004 [37], only drinking water can be used for carcasses, while lactic acid is also allowed for beef carcasses. The European Commission has not approved phage products for reducing bacteria in ready-to-eat (RTE) foods and have limited their use to processing aids that aim to kill pathogens on carcasses. This is partly due to concerns that phages could replace, rather than complement, hygiene measures. In contrast, several countries (e.g., United States, Brazil, The Netherlands, Israel, Canada, Switzerland, Australia, and New Zealand) have authorized phage preparations as auxiliary tools for controlling foodborne pathogens. Commercial products are available from companies such as PhageGuard (Micreos Food Safety), Intralytix, and Phagelux, targeting pathogens including *Salmonella* spp., *Listeria monocytogenes*, *Escherichia coli*, and *Shigella* spp. (Table S1).

### 2.4. Phage Bioproducts for Work and Production Surfaces (Food Facilities)

Substances used to remove microbial contamination from work surfaces, equipment, vessels, and supply lines are subject to biocidal product regulations, including provisions on market placement [38]. Using phages to target biofilms on surfaces that come into contact with food is seen as a promising approach, but it has not yet been approved in the EU [39]. In contrast, the FDA may authorize the use of phages on food-contact surfaces under the Food Contact Substance (FCS) designation, which may not require labeling [40]. Notably, the same phage preparation may be classified as a processing aid or as a food additive depending on whether phages remain in the food and on the intended purpose of application. A risk-based regulatory system could connect the requirements for documentation and labeling, with the public health relevance [18].

### 2.5. Classification of Endolysins/Lysins

Endolysins (lysins) are enzymes synthesized by bacteriophages during late infection stages that kill bacteria by degrading peptidoglycan in the cell wall. Compared with whole bacteriophages, they may display broader activity against multiple bacterial strains or species, and resistance has not yet been widely reported [21]. However, their use in food applications has certain limitations. Food matrices can diminish bactericidal activity and narrow the range of effective hosts, and they typically show limited effectiveness against Gram-negative bacteria. Thus, their application must be validated under relevant conditions such as pH, temperature, and food composition. Thermostability is a key determinant of efficacy, and engineered endolysins have been developed to improve stability [21].

From a regulatory perspective, their enzymatic nature, defined composition, and mechanism of action distinguish them from whole phage preparations, suggesting that they may be more appropriately evaluated under frameworks similar to food enzymes or protein-based additives, with distinct safety and efficacy requirements. To date, Nomad Bioscience GmbH has submitted a GRAS notice (GRN 000802) to the FDA for an endolysin preparation, including safety data and demonstrated activity against *Clostridium perfringens* in laboratory and cooked meat models. Criteria for activity, stability, and exposure levels were defined, with an estimated dietary exposure of 2.6 mg/person/day at an application rate of 10 mg/kg [41].

Taken together, Figure 1 shows that there is no global agreement on how to classify and approve bacteriophage-based products. Different countries have different rules. Some use clear categories like GRAS or processing aids, while others allow products to be approved on a case-by-case basis. This variety makes it hard to classify and label products, creating confusion for developers who want to get approval to sell their products in different regions. Because of these differing regulations, it becomes challenging to widely implement phage technologies. This highlights the need for a coordinated international approach.

South American countries face the challenge of deciding whether to adopt foreign regulatory frameworks as a reference or to develop their own local regulations, with the limitations and risks that both options involve. In this context, important challenges still remain, especially the difficulty of classifying phage-based bioproducts according to their intended use, the absence of harmonized criteria for health authorization, labeling, and documentation, and the lack of international consensus on whether these products should be regulated as processing aids, food additives, biocides, or through case-by-case authorizations. In this sense, one of the main gaps in South American countries is the lack of clear labeling criteria, both for phage applications at pre-harvest stages and for foods treated with these bioproducts. Since there is no defined regulatory classification, health authorities face difficulties in assigning a risk level to the product, its intended use, and the phage application process. This uncertainty limits technical assessment, traceability, and the safe implementation of phage-based technologies in the food systems of the region.

For bacteriophage-based products, it is important to distinguish between related but non-equivalent regulatory concepts, including health authorization, marketing or commercial authorization, free-sale certification, and product classification. Product classification is the initial regulatory step that determines the legal category under which a phage-based product will be evaluated, such as food additive, processing aid, veterinary medicinal product, feed additive, biocontrol agent, disinfectant, or biological product. This classification defines the applicable safety, quality, efficacy, labeling, and monitoring requirements. Health authorization refers to the technical and sanitary evaluation by the competent authority to determine whether the product is safe and suitable for its intended use. Marketing or commercial authorization, in contrast, allows the product to be placed on the market once the required regulatory conditions have been met. Free-sale certification is a different instrument: it usually certifies that a product is legally manufactured and/or commercialized in a given country, often to support export or registration processes in another jurisdiction, but it does not necessarily replace a full safety or efficacy assessment by the importing country. In the case of bacteriophage products, confusion among these concepts can create regulatory uncertainty, particularly when the same phage preparation could be interpreted differently depending on its intended use, target matrix, claim, and application point along the farm-to-fork continuum.

## 3. Challenges for Phage Use in Farm-to-Fork Food Production: Implications for South America

Phages are biological agents that can be effective and safe, but this depends on the host environment, the type of food, and how they are applied from the farm to the table. To use them successfully, we need to tackle several related scientific, technological, and regulatory issues.

### 3.1. Verifying Specificity and Persistence in Target Bacterial Populations

Phage specificity is a defining property that must be evaluated in relation to the target bacterial species, the food matrix, and the stage in the production chain where phages are implemented (e.g., *Salmonella* and *Campylobacter* in raw poultry products) [42]. This specificity is influenced by receptor availability, bacterial physiological state, and environmental conditions, all of which may vary substantially between production systems.

Several phage-based products are currently available on the market, but these products were developed and tested in areas where the types of bacteria differ from those found in local production systems. As a result, their efficacy under local conditions may be reduced, particularly when host range does not adequately cover the diversity of circulating strains. This shows the need to create and test phage cocktails that are specifically designed for different host groups and production settings. The cocktails should be designed based on data from local strains and real-life conditions [42].

Current regulatory frameworks have largely relied on the authorization of individual products based on laboratory assays and limited field trials. However, these approaches may not capture the variability encountered under industrial conditions, including differences in temperature, pH, organic load, and microbial community structure. Consequently, such evidence is often insufficient to ensure sustained effectiveness over time [18,38,43,44]. In contrast, human phage therapy has moved toward adaptive and personalized strategies, including magistral preparations, which allow continuous adjustment to evolving bacterial populations [45]. Translating similar adaptive principles into food systems remains a key challenge. Therefore, regulatory frameworks should require that phage-based treatments are tested against locally circulating bacterial populations to ensure the sustained effectiveness of these products.

### 3.2. Addressing the Emergence of Phage-Resistant Bacteria

The emergence of phage-resistant bacteria is an inherent risk in phage treatments. Resistance can develop when phages live alongside active bacteria over time. This can happen during large-scale production or when using them in complex environments [43]. Mechanisms of resistance include receptor modification or loss [46], restriction–modification systems, CRISPR-Cas immunity, and abortive infection systems. The use of phage cocktails (typically ≥3 phages) is a common strategy to reduce the probability of resistance emergence by simultaneously targeting multiple bacterial receptors [18,38]. However, even under these conditions, resistant or partially susceptible subpopulations may persist and compromise efficacy, particularly when application parameters such as multiplicity of infection (MOI), contact time, and distribution within the food matrix are suboptimal [38,43,44].

Therefore, maintaining effectiveness requires continuous monitoring of phage performance and periodic updating of phage cocktails in response to shifts in bacterial populations. This dynamic approach challenges conventional regulatory models, which are typically designed for unchanging products. Regulatory frameworks must therefore define mechanisms for approving modifications to phage formulations while ensuring consistency and safety [18]. In this context, the European Medicines Agency (EMA) [32], concept paper proposes phage-bank-based approaches that allow controlled updates to authorized products and provide guidance on how such changes should be documented and evaluated.

In this regard, the CVMP/EMA “Concept paper on quality, safety and efficacy of bacteriophages as veterinary medicines” [32], is particularly relevant, because it supports using a phage bank to update approved phage treatments by adding new phages. It also highlights the need to reflect these updates in the Summary of Product Characteristics (SPC) and suggests including a glossary to avoid confusion. EMA also explicitly recognizes veterinary phages as novel therapies aligned with Regulation (EU) 2019/6 [32], and notes regulatory complexity because: (i) host range is narrow and resistance can emerge rapidly; (ii) “pharmacology” is dynamic (amplification depends on susceptible bacteria and immune responses); and (iii) many products will require cocktails with a variable qualitative–quantitative composition that must be updated over time. The document sets quality requirements for a master seed and seed stock system. It stresses confirming lytic phages from phage banks and screening for antimicrobial resistance genes and virulence factors. It also promotes a risk-based approach for different contexts, such as companion animals versus production animals and prophylactic versus therapeutic treatments. In addition, EMA specifies the need for studies with the target animals, definition of minimum effective dose, posology/duration, and assessment of resistance development, and crucially includes guidance on how updates will be evaluated [32].

### 3.3. Demonstrating Long-Term Residual Effects and Monitoring

The evaluation of phage-based bioproducts should include both post-market monitoring and long-term assessments. This requires robust quality systems incorporating traceability, genomic characterization to exclude undesirable genes (e.g., virulence or antimicrobial resistance determinants), periodic verification of host range, and validation of performance under real production conditions.

Environmental considerations remain an area of uncertainty. Although doses used in food applications (≈10^8^ PFU/g) are small relative to naturally occurring phage populations (≈4.5 × 10^22^ PFU) [18,47], repeated or large-scale applications, especially in biosanitation contexts, may influence microbial ecosystems [39]. Additional factors include phage persistence in processing environments, interactions with disinfectants, and potential effects on non-target microbial communities. Certain phages may show decreased susceptibility to widely used sanitizers, highlighting the need for integrated control strategies. These factors underline the importance of structured monitoring frameworks and adaptive management approaches, which include periodically updating phage cocktails to maintain their effectiveness.

### 3.4. Regulatory Implications of Phage Ingestion Through Treated Foods (Farm-to-Fork)

One of the main regulatory barriers to the adoption of phage-based bioproducts in food systems lies in the difficulty of assigning these biological agents to existing regulatory categories. Accordingly, the key regulatory definitions relevant to farm-to-fork phage bioproducts are summarized below. Human exposure to phages through treated foods represents an additional regulatory consideration. Available evidence indicates that oral administration of phages is generally well tolerated, although most studies are short-term and involve small sample sizes. Experimental studies have reported potential effects on intestinal permeability and inflammatory responses; however, these findings are based on limited evidence and should be interpreted cautiously [18,38]. The potential impact of phage ingestion depends strongly on their specificity. For example, pathogens such as *Listeria* and *Campylobacter* are not part of the normal microbiota, and their lysis is expected to be beneficial. In contrast, *Escherichia coli* includes commensal strains, making precise targeting essential to avoid disrupting the microbiome [48,49]. In this sense, evidence suggests that phage administration does not significantly alter commensal populations under typical conditions. However, the lack of longitudinal studies remains a critical gap for translating these findings into a robust regulatory criterion for phage-treated foods [16].

### 3.5. Regulatory Particularities for Phage Bioproducts for Plant Use

Phage applications in crop production introduce additional complexity, as they frequently involve release into open environments (e.g., foliar sprays, seed treatments, or irrigation systems) [50]. These applications raise specific considerations related to environmental persistence, UV and desiccation stability, exposure of non-target bacteria (e.g., phyllosphere microbiomes), and variability in efficacy under field conditions [51].

Regulations for using phages as biopesticides exist in the U.S. and Canada. These rules include testing for effectiveness, assessing environmental risks, and checking for residues [52,53].

In South America, however, plant applications are typically regulated under agricultural or phytosanitary legislation rather than food safety frameworks, resulting in a lack of coordination between the regulations associated with food production and zootechnical product [33]. So, a unified approach should include specific goals for different crops, like reducing disease rates and ensuring quality attributes such as potency and safety. It also needs systems to manage lower susceptibility and allow controlled updates to phage formulations. This is particularly relevant given that bacterial crop diseases have historically driven the use of antibiotics and copper-based antimicrobials, contributing to environmental accumulation and resistance selection [51].

In summary, phage biocontrol is a focused alternative that has approval in places outside South America. To use it more widely, we need clear rules that combine plant protection with food safety, all within a One Health approach.

## 4. Scientific and Technological Development in the Region Provides an Incentive for Phage Development in South America

### 4.1. Scientific Development

A bibliographic search was conducted in PubMed/MEDLINE to identify recent evidence on the description and characterization of bacteriophages and phage-derived technologies with potential application in South American food systems. The search strategy combined the terms “phage” and “bacteriophage” with regional (“South America”) and country-specific filters and also included a food-focused sub-strategy incorporating the term “food” (Table 1). To further characterize regional scientific development in bacteriophages from a farm-to-fork perspective, and its potential relationship with applicability and commercialization, an exploratory bibliometric sweep was additionally performed using country-specific PubMed queries with the syntax “phage AND <country>” for Argentina, Bolivia, Brazil, Chile, Colombia, Ecuador, Paraguay, Peru, Uruguay, and Venezuela. Search results were exported as CSV files, merged into a single database, and deduplicated across overlapping queries using PMID to estimate unique regional counts (Supplementary Material S1).

Overall, the combined queries retrieved 1205 records. Inclusion criteria comprised original studies published in English or Spanish during the last 5 years, with complete and available abstracts. Exclusion criteria comprised studies not related to phage isolation, description, characterization, or phage-derived technologies; studies addressing only tangential phage uses without characterization; and non-primary or non-peer-reviewed publications, including reviews, protocols, editorials, editor’s notes, books, opinion papers, conference abstracts, and preprints. After screening and duplicate removal, a curated corpus of 251 unique articles considered useful for the analysis was consolidated (Table 1). After this initial classification, a title-based filter was applied to identify studies specifically related to farm-to-fork applications, resulting in 100 selected records. This approach intentionally enriched the dataset toward applied evidence across the production chain, including primary production, food processing, food safety, environmental interfaces, and phage-based control strategies. The resulting dataset shows that regionally relevant evidence supporting farm-to-fork interventions and surveillance is available in South America, although it is unevenly distributed across countries. This uneven distribution has direct implications for comparing research maturity, international visibility, and the capacity of each country to generate performance dossiers, regulatory evidence, and field validation data for future phage-based applications. Records were considered relevant to the farm-to-fork continuum when they met at least one of six predefined selection criteria: C1, food-phage, including studies involving phages associated with foods or food-related matrices, such as fruits, drinking water, chicken meat, lettuce, raw milk, cheese, coffee plants, and fresh vegetables; C2, food system-phage, including studies involving phages in food production systems, such as swine, poultry, salmon, cattle, dairy production, stingless bees, laying hens, bovine mastitis, and bivalve mollusks; C3, foodborne pathogen-phage, including studies involving phages targeting foodborne pathogens, such as *Salmonella* spp., *E. coli*, *Listeria monocytogenes*, *Pseudomonas aeruginosa*, *Staphylococcus aureus*, and *Shigella flexneri*; C4, food quality-related bacteria, including studies involving bacteria associated with food spoilage or food quality, such as *Pseudomonas fluorescens* and *Serratia* spp.; C5, plant/animal pathogen-phage, including studies involving phages targeting plant or animal pathogens relevant to food production, such as *Vibrio anguillarum*, *Piscirickettsia salmonis*, *Erwinia* spp., *Pseudomonas syringae*, kiwifruit bacterial canker, *Xanthomonas arboricola*, *Flavobacterium psychrophilum*, and *Ralstonia* spp.; and C6, other food-system-related phage studies, including records addressing food-grade additives, food packaging, crAssphage, *Rhizobium* spp., jumbo phages, or widespread phages with potential relevance to food systems or phage ecology. Within the curated set of farm-to-fork-focused articles, the analysis showed that research on bacteriophages in South America is growing and is focused on practical uses throughout the food, veterinary or zootechnical solutions [33,54,55].

This includes studying environmental sources, farming, food safety, animal health, and what happens after harvest (Thematically, the most frequent studies are associated with phage isolation, genomic and biological characterization, and the evaluation of lytic activity against priority bacterial pathogens, particularly *Salmonella enterica* [56,57,58,59], *Escherichia coli* [60,61], *Pseudomonas* spp. [62,63,64], *Staphylococcus aureus* [65,66,67], *Xanthomonas* spp. [68,69,70], *Ralstonia solanacearum* [71,72], and aquatic pathogens such as *Flavobacterium psychrophilum* [73], and *Vibrio* spp. [74,75], (Supplementary Material S1). A second relevant group of studies corresponds to applied biocontrol, especially in poultry, dairy, aquaculture, horticultural, and agricultural systems, This text covers the use of phage cocktails to control *Salmonella* in chicken meat [57]. It also addresses biofilm management, mastitis-related *Staphylococcus aureus* [66], and plant diseases in coffee [76], tomatoes [77], walnuts [69], and kiwifruit [63], as well as effectiveness in fish larvae and live feed [75]. In parallel, many studies focus on environmental monitoring and examining wastewater, rivers, drinking water, and marine and freshwater environments. Researchers use phages as indicators, e.g., MS2 or crAssphage [78,79,80] to measure microbial contamination, track viral persistence, and assess the effectiveness of sanitation. Another recurring theme is developing technologies from or using phages, such as endolysins [81], edible coatings [82], and food packaging [83]. It also includes detection tools like qPCR and TaqMan, as well as phage-based diagnostics, and strategies to improve stability and use in real conditions [84].

**Figure 2:**
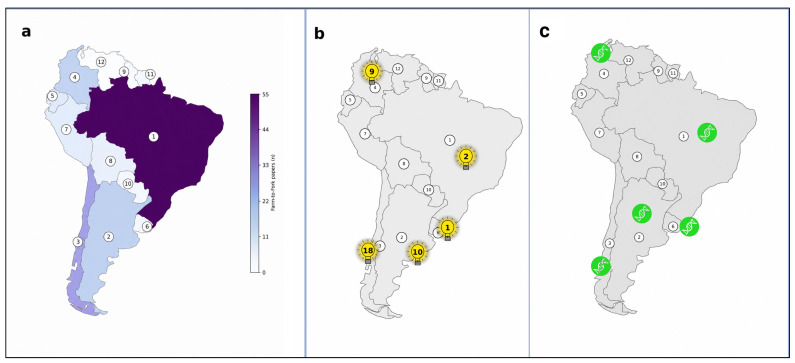
The figure presents three South America maps (panels **a**–**c**) summarizing complementary phage-related indicators across the region. (**a**) shows a choropleth of the number of farm-to-fork papers per country (color intensity by counts). (**b**) displays the number of patent inventions by country using lightbulb icons (shown only for countries with values > 0), (**c**) summarizes the presence of relevant biotechnology companies using a DNA icon by company count). Countries are identified by the numbered circles shown on each map: 1. Brazil; 2. Argentina; 3. Chile; 4. Colombia; 5. Ecuador; 6. Uruguay; 7. Perú; 8. Bolivia; 9. Guyana; 10. Paraguay; 11. Suriname; 12. Venezuela. Figure Created in https://BioRender.com.

At the country level, the dataset shows a markedly uneven distribution of farm-to-fork bacteriophage research across South America. As illustrated in Figure 2a, Brazil clearly dominates regional scientific production, with the highest number of publications (*n* = 55), followed by Chile (*n* = 21) and Argentina (*n* = 10). Colombia shows a lower but visible contribution (*n* = 5), whereas Bolivia and Ecuador present limited scientific output (*n* = 2 each), and Uruguay shows only one selected publication. In contrast, countries such as Peru, Paraguay, Venezuela, Guyana, and Suriname were not represented in the selected dataset. This pattern indicates that farm-to-fork phage research in South America is concentrated in a small group of countries, particularly Brazil, Chile, and Argentina. The thematic scope of these studies includes pathogen biocontrol, genomic characterization, biofilm reduction, environmental monitoring, food safety applications, phage formulation, and the development of technologies for agricultural and food-chain use. Overall, the regional scientific production supports the existence of a relevant applied research base for bacteriophage-based strategies; however, this capacity remains unevenly distributed among countries and sectors.

### 4.2. Innovation Ecosystems and Intellectual Property

In South America, we can assess how phage applications are advancing in food systems by examining biotechnology initiatives and patents in the region. To obtain a clear assessment, we explored patents related to this topic using WIPO Patentscope, Espacenet (EPO), and Google Patents Supplementary Material S2.

The search strategy combined a free-text phage block [FP: (phage OR bacteriophage)] with a document country code filter (PCN), using ISO-3166 alpha-2 codes for South America: BR, CL, CO, AR, EC, UY, PE, VE, BO, and PY (e.g., FP AND PCN: (BR) for Brazil; used in the same way to the other countries). Results from each platform were consolidated and cleaned to remove duplicates and are presented in Supplementary Material S2.

Based on these data, protection of phage-based products and associated processes has expanded across South American countries emerging as key hubs for intellectual property activity. These include Patent Cooperation Treaty (PCT) filings and national and international applications (Figure 2b). This analysis shows that patent-related signals for bacteriophage technologies are emerging unevenly across South America, with activity concentrated in a limited number of countries. Chile and Argentina showed the highest number of patent-related results, followed by Colombia, Brazil, and Uruguay, whereas Bolivia, Ecuador, Venezuela, Paraguay, and Peru showed no results under the applied search strategy (Figure 2b). This uneven distribution suggests that innovation and commercialization pathways for bacteriophage-based technologies remain fragmented across the region. From a broader policy perspective, Brazil and Chile provide relevant examples of how patent regulation may intersect with sustainable development goals. In a recent analysis of patent regulation in Latin America, Portes et al., [85] applied Qualitative Comparative Analysis (QCA), a comparative method used to identify combinations of conditions associated with a given policy outcome, and identified both countries as cases where policy integration for sustainable development was enabled through a configuration involving lower dependence on US trade and greater civil society access to policymaking. Although this evidence is not specific to bacteriophages, it supports the interpretation that intellectual property governance, policy space, civil society participation, and technology transfer frameworks may shape the regional capacity to translate emerging biotechnologies into regulated and competitive markets. However, it is interesting to note that, despite this broader policy context, Brazil showed only two patent results for Brazilian-origin bacteriophage biotechnology products under the applied search strategy. This contrast suggests that enabling patent policy conditions do not necessarily translate directly into sector-specific patent activity, particularly in emerging technological areas such as phage-based bioproducts. This is also consistent with recent discussions highlighting biotechnology research security and international technology transfer regimes as key challenges for Latin America’s innovation systems. In this context, biotechnology research security refers not only to biosafety and biosecurity concerns, but also to the capacity of countries to protect, regulate, and responsibly manage emerging biotechnologies while avoiding technological dependency. Similarly, international technology transfer regimes influence whether locally generated knowledge can be converted into accessible applications, protected intellectual property, and scalable products. For bacteriophage-based technologies, these issues are particularly relevant because their translation requires coordinated frameworks for intellectual property protection, regulatory evaluation, quality standards, and regional collaboration. Therefore, the fragmented patent-related signals observed in South America may reflect not only differences in scientific productivity, but also broader asymmetries in institutional capacity, technology transfer infrastructure, innovation governance, and sector-specific translational capacity across the region (Bacelo and Fioressi, 2025 [86]; Correa and Beneke, 2024 [87]).

South America is seeing the growth of initiatives that use phages, especially in agri-food systems. For example, the Chilean company PhageLab (https://phage-lab.com/) has secured significant funding and is expanding in the region, reflecting growing investment in this sector [88]. More investors and intellectual property actors are joining in, highlighting the need for stronger rules that support innovation and commercialization. In Brazil, industrial participation is reflected in the availability of commercial products such as BAFASAL-PRO (https://proteonpharma.com/es/proteon-pharmaceuticals-enhances-portfolio-with-bafasal-pro-registration-in-brazil/ accessed on 15 January 2026), used for *Salmonella* control in poultry. This indicates the presence of a more advanced market environment that is already addressing issues related to registration pathways, quality attributes, and efficacy standards. In parallel, local ventures focused on phage-based solutions for livestock production, such as Karaja Biosciences (https://pesquisaparainovacao.fapesp.br/empresa_brasileira_desenvolve_alternativa_sustentavel_a_antibioticos_para_a_pecuaria/3699 accessed on 15 January 2026), further support the expansion and diversification of the private sector. Regarding Colombia and Uruguay, initiatives such as SciPhage (https://biointropic.com/sciphage-y-bacteriofagos/ accessed on15 January 2026) and Kinzbio (https://www.kinzbio.com/, accessed on15 January 2026), respectively, illustrate how different countries can develop models associated with different areas, such as agri-food/One Health and human clinical applications. Although Kinzbio is not strictly focused on food systems, its inclusion reflects the broader regional technical capacity and knowledge in the matter (Figure 2c). These findings show that South America is establishing a strong base for science and innovation in bacteriophage use. However, ongoing regulatory uncertainty at national and regional levels is still an obstacle to the sector.

### 4.3. Challenges for Deploying Phage-Based Bioproducts

As highlighted above, South America 12. Venezuelastantial scientific and biotechnological development in bacteriophages; however, there is still a clear need to advance in their application and commercialization. The current commercialization of bacteriophage-based bioproducts in the region reflects an incipient interest but concentrated in a limited number of countries and primarily oriented toward animal production systems. The identified products primarily target livestock and focus on key pathogens that affect animal health and productivity, particularly *Salmonella* and harmful *E. coli* [16,59,89]. This approach addresses urgent issues with clear regulatory paths. Chile is becoming a key center for development and technology transfer in the region, and as such, Chilean solutions are sold and used in other countries, especially Brazil. In this regard, companies like PhageLab play an important role in connecting research and development (R&D) with industrial use. Brazil, in turn, represents a key market for scaling, where products have achieved registration and distribution through more consolidated commercial channels. Overall, the evidence summarized in Table S1 indicates that commercialization remains uneven in the region. The presence of actors with implementation capacity, who combine validated phage cocktails, technical support, and field pilots, appears to be a decisive factor in the successful adoption of this technology. It is expected that advancing harmonized regulations in the region will stimulate the development of this technology in other countries on the continent. However, approval for selling phage-based products often does not match health regulations that ensure safety for food producers and consumers, and progress in establishing clear labeling requirements is also limited. Although some products have received national or multi-country approvals (Table S1), health authorities are hesitant to grant broader approvals, leading to uncertainty for food producers and customers. This uncertainty is further amplified under stricter regulatory environments such as the European Union, where systems like TRACES require full traceability [90]. Furthermore, companies that export food products, especially to the European Union, face challenges when there are no clear national or regional regulations. Since the general use of bacteriophages is not approved in the EU, some companies may adopt conservative strategies to open new markets.

In practical production settings, several technical constraints have been reported that may limit the consistent performance of phage-based applications. These include reduced stability during storage and transport, uneven distribution across heterogeneous food matrices, and variability in efficacy due to environmental factors such as temperature, pH, and organic load. In addition, scaling up application protocols from laboratory to industrial conditions introduces challenges related to formulation, delivery systems, and process integration. While these constraints are recognized across different contexts, systematic documentation under South American production conditions remains limited, highlighting the need for region-specific validation studies.

Taken together, these findings suggest that South America is no longer constrained by a lack of scientific capacity or technological initiative but rather by the absence of coherent and harmonized regulatory pathways capable of transforming emerging phage innovations into safe, scalable, and internationally competitive farm-to-fork solutions.

## 5. Current Status of Phage Bioproducts in South America and the Need to Develop General Health Approval Standards

Health authority approvals for phage applications in agricultural systems issued by international regulators (Table S1) provide valuable precedents for South American countries, offering established models that local regulatory authorities can follow. The increasing problem of antimicrobial resistance [7,15,23,24], together with the importance of agri-food exports for the region, helps explain the rapid growth of biotechnology companies and phage-based bioproducts (Table S1). However, the regulatory framework has not kept pace with this expansion. Despite the existence of several products with marketing authorization, there are still no comprehensive health authorization frameworks that enable the broad regulation, monitoring, and surveillance of their use. This gap limits the feasibility of widespread implementation in local agricultural systems and constrains market adoption, largely due to the perceived risks associated with their use [91]. These perceptions are further reinforced when phage-based products are not explicitly integrated into food production regulations, generating uncertainty among both producers and consumers. Although several products have marketing approval, there are no clear health regulations for their use. This lack of regulation hinders their widespread implementation in local farming and limits market acceptance due to risk concerns, increasing uncertainty for farmers and consumers. In addition, this regulatory gap complicates implementation for agri-food companies engaged in export markets [90]. For example, poultry meat exports to the European Union face restrictions on the use of decontamination agents during processing, and since the generalized use of phages has not been authorized, these companies are forced to follow a case-by-case approval within the EU framework [90].

From a regional perspective, the expected outcome would be the development of a harmonized framework capable of defining product categories according to the step at which they are to be used (pre-harvest and post-harvest), the matrix (animal, food, surfaces, water, and effluents), and the intended claims. In addition, such a framework should establish universal requirements for genomic safety and product quality (purity, potency, stability). Together, these elements would reduce uncertainty for innovators, enhance consumer protection, and support the long-term sustainability of regional biotechnology initiatives.

## 6. Future Perspectives

Recent studies indicate that AMR mitigation in the region still depends on stronger multisectoral One Health coordination, standardized surveillance methods, and comparable reporting systems across human, animal, food, and environmental sectors [92,93,94]. This is particularly relevant because regional evidence on foodborne pathogens such as nontyphoidal *Salmonella* remains heterogeneous in terms of prevalence, serovar distribution, genomic diversity, and AMR profiles [95,96], while environmental reservoirs, especially water, remain insufficiently integrated into AMR surveillance frameworks [97]. Additional regional analyses on antimicrobial consumption and carbapenemase-producing bacteria further highlight the need for standardized methodologies, laboratory protocols, and regional data-sharing networks to reduce antimicrobial pressure and improve public health responses [98,99]. In this context, phage-based bioproducts require not only regulatory classification, but also agreed criteria for genomic safety, manufacturing quality, matrix-specific efficacy, post-market monitoring, and phage-resistance surveillance. Given the heterogeneity in the production and development of phage bioproducts in South America, their long-term adoption and sustainability in the region are still uncertain. This uncertainty is influenced not only by technological factors but also by perceptions among producers and consumers. In turn, this limits the feasibility of multicenter studies capable of generating comparable cross-country data, which are essential to support the scalability and exportability of phage-based bioproducts in both local and international markets. As a consequence, these constraints may reduce both public health impact and the potential contribution of phage technologies to agricultural development.

Regional alignment is therefore particularly important. South American countries share production models, exposure to similar hazards, and strong commercial interdependence in agri-food systems. In this context, the development of clear governance frameworks for phage applications should be understood as enabling infrastructure. Such frameworks would strengthen food safety by anchoring product claims to validated endpoints, support international trade by reducing discrepancies and increasing transparency, and promote innovation by lowering uncertainty for investors and producers.

A key pathway to advance this agenda is the submission of a proposal for new work within the Codex Alimentarius framework, so that phage bioproducts are formally addressed by an appropriate Codex committee [36]. The Codex Alimentarius Commission, established under the FAO/WHO Joint Programme, is the international body responsible for developing food standards, guidelines, and codes of practice to protect consumer health and ensure fair practices in the food trade [36]. The Codex Alimentarius currently includes 189 members (188 countries and the European Union), with active participation from South American countries [36].

**Figure 3:**
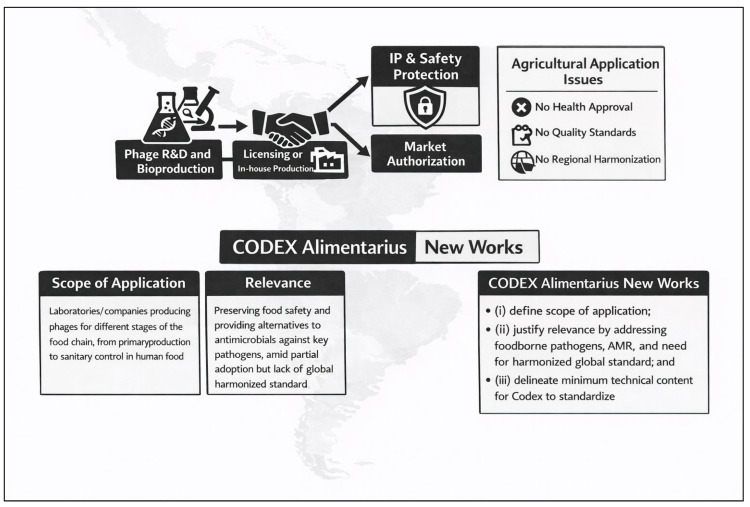
Regulatory gaps for phage-based bioproducts in South America and the rationale for a Codex “New Work”. The upper panel illustrates the typical development-to-market pathway for phage bioproducts in the region, phage research and development (R&D), licensing or in-house manufacturing, intellectual property (IP) and safety protection, and market authorization, and summarizes the main barriers currently limiting agricultural deployment, including the lack of health approvals, the absence of country-level quality standards, and limited regional harmonization. The lower panel presents the proposed Codex Alimentarius-New Work, highlighting its scope and relevance and the minimum technical elements recommended for standardization to enable safe, effective, and internationally consistent farm-to-fork applications.

As illustrated in Figure 3, the upper panel depicts a fragmented development-to-market pathway, from research and development to commercialization, where key bottlenecks, particularly the lack of health approvals and harmonized standards, constrain large-scale agricultural deployment. In contrast, the lower panel outlines a proposed Codex “New Work” framework [100], defining the minimum technical elements required to support consistent evaluation and implementation across the farm-to-fork continuum. Together, the figure highlights the need for coordinated international guidance to bridge this gap and enable safe and scalable adoption.

In the absence of clear and harmonized rules, products may either remain fixed in outdated formulations or be updated without transparent criteria to ensure comparability in terms of safety and efficacy. A similar challenge applies to the broader phage innovation ecosystem: while some countries may be capable of generating multicenter validation data, others may first require minimum regulatory definitions, standardized methodologies, and baseline datasets before scaling farm-to-fork solutions [16,18,47,100]. Moreover, the lack of national regulatory frameworks prevents competent authorities from consistently assessing and comparing product quality. At the regional level, the absence of harmonized guidelines further limits the possibility of establishing equivalent product classifications (e.g., food additive, biocide, or processing aid) across countries. This has direct implications in a context where raw materials and food products circulate extensively within South America, highlighting the need for coordinated regulatory approaches that support both internal market integration and international competitiveness.

Although there are currently no Codex standards specifically addressing phage bioproducts, their inclusion would complement existing guidance on food safety and the safe reuse of inputs. For example, during the 48th session of the Codex Committee on Food Hygiene (CCFH) in 2016, the need to further investigate bacteriophages as a control strategy for *Salmonella* in poultry was highlighted [101]. This interest has continued in recent scientific literature, suggesting that future revisions of Codex guidelines should formally consider this technology. Additional relevant precedents include the review of CXG 100-2023 (water in foods) and ongoing initiatives related to biocontrol and microbiological risk reduction [102]. Within the CCFH, particularly in the context of revising the Guidelines for the Control of *Campylobacter* and *Salmonella* in chicken meat (CXG 78-2011) [103], alternative interventions to reduce bacterial loads have been evaluated, with phages identified as potential tools contingent upon demonstrated safety, efficacy, and regulatory acceptance.

In this context, a Codex-New work proposal focused on phage bioproducts could address a critical gap by establishing guidelines for the production and application of bacteriophages along the food chain [16,18,47,100]. Such a proposal would define a clear scope for laboratories and companies operating at different stages of the food system (e.g., primary production and sanitary control), provide a justification based on the burden of foodborne pathogens and the need for alternatives under antimicrobial resistance (AMR) pressure, and outline minimum technical elements requiring standardization [16,18,47,100]. These elements would include definitions and classification criteria, characterization requirements (including genomic assessment), critical quality attributes (e.g., purity, concentration, shelf life, and storage conditions), production and formulation standards, labeling considerations, and intended uses [18].

The establishment of such guidelines would support the safe and effective deployment of phage-based bioproducts while facilitating regulatory alignment across countries. In the absence of international standards, adoption remains fragmented, creating uncertainty, limiting comparability of regulatory dossiers, and potentially generating technical barriers to trade [90]. Conversely, harmonized guidance would improve regulatory predictability, support market access, and strengthen the integration of phage technologies into existing food safety systems.

Ultimately, advancing toward coordinated governance of phage bioproducts represents a necessary step to transition from fragmented adoption to structured implementation. By aligning scientific evidence, regulatory frameworks, and market needs, South America has the opportunity to position bacteriophage-based solutions as a scalable, farm-to-fork strategy contributing to food safety, antimicrobial resistance mitigation, and sustainable agri-food systems.

## 7. Conclusions

Phage-based bioproducts have strong potential to improve food safety and reduce antimicrobial pressure across the farm-to-fork continuum in South America. However, their sustainable and large-scale adoption will depend on addressing critical regulatory gaps. This requires moving beyond fragmented, case-by-case authorizations toward a harmonized, risk-based framework that incorporates demonstrated specificity against locally relevant bacterial populations, monitoring systems, transparent mechanisms for updating phage cocktails, and consistent criteria for product classification and labeling.

Although the region is showing growing scientific, technological, and innovation capacity, commercialization remains uneven and broader health authorization frameworks are still lacking. This continues to limit confidence among producers, consumers, and export-oriented sectors. Advancing harmonized guidance should therefore be considered a regional priority. In practical terms, initial steps may include the development of interim national guidelines, the definition of minimum quality and genomic safety criteria, and the implementation of pilot regulatory frameworks to support controlled deployment. Ideally, these efforts should converge in a Codex-New work capable of supporting the safe, scalable, and internationally consistent implementation of phage-based bioproducts at both regional and global levels.

## Figures and Tables

**Table 1:** Search strategy and selection of bacteriophage-related studies relevant to farm-to-fork applications in South America.

Search Block/Region	Search Strings Included	Records Retrieved (*n*)	Potentially Relevant After Screening (*n*)	Farm-to-Fork Selected Records (*n*)	Selection Criteria Applied
South America	(bacteriophage) AND (South America); (phage) AND (South America); ((bacteriophage) AND (food)) AND (South America); ((phage) AND (food)) AND (South America)	166	11	4	C1–C6
Brazil	(phage) AND (Brazil); (bacteriophage) AND (Brazil)	577	145	55	C1–C6
Colombia	(phage) AND (Colombia); (bacteriophage) AND (Colombia)	121	18	5	C1–C6
Chile	(phage) AND (Chile); (bacteriophage) AND (Chile)	136	33	21	C1–C6
Argentina	(phage) AND (Argentina); (bacteriophage) AND (Argentina) *	96	30	10	C1–C6
Ecuador	(phage) AND (Ecuador); (bacteriophage) AND (Ecuador)	32	5	2	C1–C6
Bolivia	(phage) AND (Bolivia); (bacteriophage) AND (Bolivia)	9	1	1	C1–C6
Bolivia/Argentina	Cross-country record identified during screening	—	—	1	C1–C6
Uruguay	(phage) AND (Uruguay); (bacteriophage) AND (Uruguay)	23	5	1	C1–C6
Peru	(phage) AND (Peru); (bacteriophage) AND (Peru)	17	2	0	C1–C6
Venezuela	(phage) AND (Venezuela); (bacteriophage) AND (Venezuela)	20	0	0	C1–C6
Paraguay	(phage) AND (Paraguay); (bacteriophage) AND (Paraguay)	8	1	0	C1–C6
Total		1205	251	100	

(*) Selected scientific articles that were written in more than one country. Note: Records were classified as farm-to-fork relevant when they addressed bacteriophages associated with food matrices, food production systems, foodborne pathogens, food quality-related bacteria, plant or animal pathogens relevant to food systems, or other applications connected to food-grade additives, packaging, or phage ecology. The total number of records retrieved corresponds to the sum of all search strings and may include duplicates. Final selected records correspond to studies retained after relevance screening according to the predefined farm-to-fork criteria.

## Data Availability

The data supporting this review were obtained from publicly available information sources, including PubMed/MEDLINE, Google Patents, WIPO Patentscope, and Espacenet from the European Patent Office (EPO). The curated datasets and analytical synthesis generated for this manuscript are provided in the Supplementary Materials. No new experimental data were generated in this study.

## Supplementary Materials

The following supporting information can be downloaded at https://www.mdpi.com/article/10.3390/foods15112031/s1: Supplementary Material S1: Bibliographic dataset supporting the scientific landscape analysis of bacteriophage applications in South America, Supplementary Material S2: Patent landscape dataset supporting the technological analysis of bacteriophage applications in South America, Table S1: International regulatory frameworks and authorization pathways for bacteriophage-based products in food and agricultural systems.
