# Supplementary material for: Regulatory Harmonization Needs for Farm-to-Fork Bacteriophage Applications in South American Food Systems
Source: Foods. 2026 Jun 5;15(11):2031. doi: 10.3390/foods15112031 (PMC13257019; doi:10.3390/foods15112031)
Supplement: Supplementary file 1 [file foods-15-02031-s001.zip › Supp_material-Table_S1.pdf]

Regulatory category	Intended use Category	Country / Region/State-regulation	Product name	Approved use/authorized target	Access link	Regulatory route clarification
Generally Recognized as Safe (GRAS)	Preharvest	**United States** Food and Drug administration (FDA) FSIS: Food Safety and Inspection Service of USDA/ Environmental Protection **United States** Agency (EPA-USA)	PhageGuard E Hides/Micreos	Processing aid by FSIS Directive 7120.1/On animals	https://phageguard.com/solutions/e-coli	Product classification/safety route: FDA GRAS notice or food-additive provision, sometimes combined with FSIS processing-aid listing. This indicates safety/use acceptance for specified matrices and is distinct from free-sale certification.
GRAS	Preharvest		*Escherichia coli* STEC/EcoShield PX™/Intralytix	GRN 834 extended/n.k./ Animal, partially also on food	https://www.intralytix.com/product/11?e=EcoShield https://www.hfpappexternal.fda.gov/scripts/fdcc/index.cfm?set=GRASNotices&id=834	Product classification/safety route: FDA GRAS notice for a specified intended use. This is not equivalent to a general marketing authorization or free-sale certificate.
GRAS	Post-harvest	**United States** Food and Drug administration (FDA)/ FSIS: Food Safety and Inspection Service of USDA	*Listeria* phage P100 (Listex™ P100)	GRAS-status *byFDA (GRN 528)/ Food additive under 21 CFR §172.785 by FDA Processing aid with FSIS Directive 7120.1/ Ham, sausages, poultry products, fish and seafood fresh and processed fruits and vegetables as an antimicrobial processing aid to control *Listeria monocytogenes* in foods (including meat/poultry)	https://hfpappexternal.fda.gov/scripts/fdcc/index.cfm?id=218&order=DESC&search=bacteriophage&set=GRASNotices&sort=Date_of_closure&startrow=1&type=basic	Product classification/safety route: FDA GRAS notice or food-additive provision, sometimes combined with FSIS processing-aid listing. This indicates safety/use acceptance for specified matrices and is distinct from free-sale certification.
GRAS			PhageGuard L™(Listex P 100)/ Micreos	GRAS status by FDA (GRN 198, GRN 218 for supplemented cocktail)/ Meat and poultry products, cheese (e.g. on the rind of hard cheeses), fish and seafood fresh and processed fruits and vegetables	https://www.phageguard.com/solutions/listeria https://hfpappexternal.fda.gov/scripts/fdcc/index.cfm?set=GRASNotices&id=218&sort=GRN_No&order=ASC&startrow=1&type=basic&search=22	Product classification/safety route: FDA GRAS notice for a specified intended use. This is not equivalent to a general marketing authorization or free-sale certificate.
GRAS			Escherichia coliO157:H7 PhageGuard E™/ Micreos	GRAS status by FDA (GRN 757) Processing aid with FSIS Directive 7120.1/ Beef carcasses, meat-trimmings, vegetables	https://phageguard.com/solutions/e-coli https://www.hfpappexternal.fda.gov/scripts/fdcc/index.cfm?set=GRASNotices&id=757	Product classification/safety route: FDA GRAS notice or food-additive provision, sometimes combined with FSIS processing-aid listing. This indicates safety/use acceptance for specified matrices and is distinct from free-sale certification.
GRAS			EcoShield™/Intralytix	GRAS status by FDA (GRN 834)/Red meat parts, trim for ground, as meat, poultry, dairy products fruits and vegetables	https://www.intralytix.com/product/2 https://www.hfpappexternal.fda.gov/scripts/fdcc/index.cfm?set=GRASNotices&id=834	Product classification/safety route: FDA GRAS notice for a specified intended use. This is not equivalent to a general marketing authorization or free-sale certificate.

GRAS			*Salmonella enterica* PhageGuard S™(Salmonelex)/Micreos	GRAS status by FDA (GRN 468, GRN 859 for supple mented cocktail). Processing aid with FSIS Directive 7120.1/ Certain pork and poultry prod- ucts; beef vegetables fresh and saltwater seafood	https://www.phageguard.com/es/soluciones/salmonela https://www.hfpappexternal.fda.gov/scripts/fdcc/index.cfm?set=GRASNotices&id=468	Product classification/safety route: FDA GRAS notice or food-additive provision, sometimes combined with FSIS processing-aid listing. This indicates safety/use acceptance for specified matrices and is distinct from free-sale certification.
GRAS			SalmoFresh™(former SalmoShield)/Intralytix	GRAS status byFDA (GRN 435) Processing aid with FSIS Di- rective 7120.1/Reduction of Salmonella on food	https://www.intralytix.com/product/3?e=SalmoFresh https://www.hfpappexternal.fda.gov/scripts/fdcc/index.cfm?set=GRASNotices&id=435	Product classification/safety route: FDA GRAS notice or food-additive provision, sometimes combined with FSIS processing-aid listing. This indicates safety/use acceptance for specified matrices and is distinct from free-sale certification.
GRAS			SalmoPro™/Phagelux	GRAS status ∗byFDA (GRN 603, GRN 752 for supple- mented cocktail)/Poultry products, on poultry sur- faces (max 108 PFU/g)	https://agriphage.com/food-safety/agriphage-salmopro/ https://www.hfpappexternal.fda.gov/scripts/fdcc/index.cfm?set=GRASNotices&id=603	Product classification/safety route: FDA GRAS notice for a specified intended use. This is not equivalent to a general marketing authorization or free-sale certificate.
GRAS			PhageFend™/Cytophage	GRAS status ∗byFDA (GRN 1163)/ Raw poultry before processing	https://cytophage.com/news/cytophage-secures-health-canada-validation-for-ovaphage-and-phagefend-products-for-use-in-food-safety/ https://www.hfpappexternal.fda.gov/scripts/fdcc/index.cfm?set=GRASNotices&id=1163	Product classification/safety route: FDA GRAS notice for a specified intended use. This is not equivalent to a general marketing authorization or free-sale certificate.
GRAS			Applied Phage Meat S2/Fink Tec GmbH	GRAS status ∗by FDA(GRN 1038)/ Raw meat, poultry, carcasses, primals, subprimals, trimmings (105 –107 PFU/g)	https://www.finktec.com/applied-phage https://www.hfpappexternal.fda.gov/scripts/fdcc/index.cfm?set=GRASNotices&id=1038	Product classification/safety route: FDA GRAS notice for a specified intended use. This is not equivalent to a general marketing authorization or free-sale certificate.
GRAS			Applied Phage Vegetable S2/Fink Tec GmbH	GRAS status ∗byFDA (GRN 1070)/ Fresh and processed fruits and vegetables	https://www.finktec.com/applied-phage https://hfpappexternal.fda.gov/scripts/fdcc/index.cfm?set=GRASNotices&id=1070&sort=GRN_No&order=DESC&startrow=1&type=advanced&search=%C2%A4%C2%A4chitosan%20chitin%C2%A4	Product classification/safety route: FDA GRAS notice for a specified intended use. This is not equivalent to a general marketing authorization or free-sale certificate.
GRAS			*S. Enteritidis* Phage Prep./Qingdao Phagepharm Bio-Tech Co Shigella spp/ShigaShield™/Intralytix	GRAS status FDA (GRN 1134) in Ground chicken /GRAS status byFDA (GRN 672) On foods susceptible to Shigella contamination as fresh produce, meat, and poultry	https://www.intralytix.com/product/8?e=ShigaShield https://hfpappexternal.fda.gov/scripts/fdcc/index.cfm?set=GRASNotices&id=1134	Product classification/safety route: FDA GRAS notice for a specified intended use. This is not equivalent to a general marketing authorization or free-sale certificate.
GRAS			*Campylobacter jejuni, C. coli Campyshield* ™/Intralytix	GRAS status by FDA (GRN 966)	https://www.intralytix.com/product/12?e=CampyShield https://hfpappexternal.fda.gov/scripts/fdcc/index.cfm?set=grasnotices&id=966	Product classification/safety route: FDA GRAS notice for a specified intended use. This is not equivalent to a general marketing authorization or free-sale certificate.

Processing aids	Pre Harvest	**Canada** (Health Canada) Environmental Protection Agency (EPA-USA)	AgriPhage – Tomato Canker (CMM) Biological Control	Registration decision document for AgriPhage-CMM (bacteriophage of *Clavibacter michiganensis* subsp. michiganensis)	Health Canada Pest Management Regulatory Agency. 2012 https://agriphage.com/product-info/cmm/	Biocontrol/product registration route for crop protection use. This should be distinguished from food-processing-aid LONO/iLONO routes.
Processing aids	Post-harvest		PhageGuard L™/Micreos: LONO	Processing aid by LONO (GRN 198) Listeria monocytogenes Surface treatment of meat, fish, cheese, RTE products	https://www.canada.ca/en/health-canada/services/food-nutrition/reports-publications/food-safety/policy-issuing-interim-letter-no-objection-ilono-food-processing.html	Processing-aid/no-objection route: Health Canada LONO or iLONO indicates no objection for the specified use. It is not the same instrument as a free-sale certificate or a broad market authorization.
			ListShield/Intralytix	Processing aid by iLONO (GRN 528) Meat, fish, poultry, plant-based RTE products		Processing-aid/no-objection route: Health Canada LONO or iLONO indicates no objection for the specified use. It is not the same instrument as a free-sale certificate or a broad market authorization.
			EcoShield™/Intralytix	Processing aid by iLONO (GRN 834)/ Escherichia coliO157:H7 Red meat parts and trim prior to grinding		Processing-aid/no-objection route: Health Canada LONO or iLONO indicates no objection for the specified use. It is not the same instrument as a free-sale certificate or a broad market authorization.
			PhageGuard S™/Micreos	Processing aid by LONO (GRN 468)/ Salmonella Poultry, meat, fish, fruit/ vegetables		Processing-aid/no-objection route: Health Canada LONO or iLONO indicates no objection for the specified use. It is not the same instrument as a free-sale certificate or a broad market authorization.
			SalmoFresh™/Intralytix	Processing aid by LONO (GRN 435)		Processing-aid/no-objection route: Health Canada LONO or iLONO indicates no objection for the specified use. It is not the same instrument as a free-sale certificate or a broad market authorization.
			PhageFend™/Cytophage	Processing aid by FDA (GRN 1163)/ Raw poultry before processing		Processing-aid/no-objection route: Health Canada LONO or iLONO indicates no objection for the specified use. It is not the same instrument as a free-sale certificate or a broad market authorization.
			OvaPhage™/Cytophage	Processing aid by LONO (no GRN)/ Surface treatment of eggs		Processing-aid/no-objection route: Health Canada LONO or iLONO indicates no objection for the specified use. It is not the same instrument as a free-sale certificate or a broad market authorization.
Processing aids	Post-harvest	**Australia /New Zealand** FSANZ approval under the Food Standards Code	P100 (Listex™ P100)	Unknown regulatory identification code/ EPA (HSNO) L. monocytogenes/approval (approved with controls) for import/release of the organism for intended processing-aid use	https://www.foodstandards.gov.au/food-standards-code/applications/applicationa1045bact4797	Processing-aid/organism-approval route: FSANZ Food Standards Code and, where applicable, New Zealand HSNO controls. This is a product- and use-specific authorization/classification route.

Processing aids			PhageGard L™/Micreos/	Processing aid (GRN 198)/ Listeria monocytogenes RTE-products		Processing-aid/organism-approval route: FSANZ Food Standards Code and, where applicable, New Zealand HSNO controls. This is a product- and use-specific authorization/classification route.
Processing aids			PhageGard S ™/Micreos	GRN 468)/ Salmonella processing aid/Raw meat and raw poultry meat	https://www.epa.govt.nz/database-search/hsno-application-register/view/APP202089/	Processing-aid/organism-approval route: FSANZ Food Standards Code and, where applicable, New Zealand HSNO controls. This is a product- and use-specific authorization/classification route.
Processing aids	Preharvest and post-harvest	**Switzerland** National processing-aid allowance (product/use-specific) under Swiss food ordinances (FDHA)	PhageGuard L™/Micreos	Processing aid (GRN 198)/ Listeria monocytogenes Cheese production	https://phageguard.com/knowledge-center/anti-listeria-solution-phageguard-l-pgl-approved-for-cheese-production-in-switzerland	National product/use-specific processing-aid allowance. This should be interpreted as a targeted authorization, not a general phage-product category.
Processing aids	Preharvest and post-harvest	**Israel** Innovation Authority (IIA)	EcoBrass®/ **GoldenEco**®/ **EcoFire**®	Unknown regulatory identification code	https://ecophage.com/news4/#:~:text=Crop%20Disease%20%2D%20EcoPhage-,Bayer%20Launches%20Israeli%20Company%20to%20Develop%20Viruses%20Against%20Crop%20Disease,bacteria%2Dhunting%20viruses%20called%20bacteriophages.	Innovation or product-development signal unless a competent-authority authorization is documented. GRAS numbers listed for products should not be interpreted as Israeli authorization without local regulatory evidence.
Processing aids	Post-harvest	**Israel** Innovation Authority (IIA)	PhageGuard L™/Micreos ListShield/Intralytix EcoShield™/Intralytix PhageGard S™/Micreos SalmoFresh™/Intralytix	(GRN 198)/ (GRN 528)/ (GRN 834)/ (GRN 468)/ (GRN 435)/ Listeria monocytogenes, Escherichia coli O157:H7, Salmonella	Multiple access links previously reported for the United States	Innovation or product-development signal unless a competent-authority authorization is documented. GRAS numbers listed for products should not be interpreted as Israeli authorization without local regulatory evidence.
Processing aids	Post-harvest and pre harvest	**Netherlands** European Food Safety Authority (EFSA)	PhageGuard L™/Micreos	Processing aid (GRN 198) Listeria monocytogenes Raw meat and poultry, cheese production	https://eur-lex.europa.eu/legal-content/EN/TXT/PDF/?uri=CELEX:62022CJ0745	EU/EEA case-by-case context: phage products are generally assessed according to intended use and legal classification. A product-specific decision should be distinguished from a general market authorization.
Processing aids	Post-harvest and pre harvest	**India** Food Safety and Standards Authority of India (FSSAI)	PhageGard S™/Micreos	Processing aid (GRN 468) *Salmonella* For food (not specified,) on food contact surfaces		Processing-aid or food-use classification should be verified with FSSAI documentation. Treat as use-specific regulatory evidence unless a formal authorization number is available.
Processing aids	Post-harvest and pre harvest	**Egypt**	PhageGard S™/Micreos	Processing aid (GRN 468)/Poultry production		Reported processing-aid/market-use route; competent-authority authorization and product classification should be verified.

Authorization upon request	Preharvest	**Brazil**<br>(Ministry of Agriculture, Livestock Farming and Supplies (MAPA)<br>By European Union(European Commission/EFSA)	BAFASAL PRO /Proteon<br>BAFACOL B /Proteon	Unknown regulatory identification code /Feed additive/ *Salmonella* Poultry feed<br>Feed additive/ *E.coli* Poultry feed	https://proteonpharma.com/es/bafasal-moves-closer-to-eu-authorisation/<br>https://food.ec.europa.eu/horizontal-topics/committees/paff-committees_en<br>https://www.planalto.gov.br/ccivil_03/_ato2023-2026/2024/lei/l15070.htm#:~:text=Disp%C3%B5e%20sobre%20a%20produ%C3%A7%C3%A3o%2C%20a,e%20os%20incentivos%20%C3%A0%20produ%C3%A7%C3%A3o	EU/EEA case-by-case context: phage products are generally assessed according to intended use and legal classification. A product-specific decision should be distinguished from a general market authorization.
Authorization upon request	Preharvest	**Brazil**<br>Ministry of Agriculture, Livestock Farming and Supplies (MAPA)	PHAGEIN Developed by PhageLab; distributed by MSD Animal Health	Unknown regulatory identification code/Oral product for neonatal calf diarrhea (ruminants)	2026/2024/lei/l15070.htm#:~:text=Disp%C3%B5e%20sobre%20a%20produ%C3%A7%C3%A3o%2C%20a,e%20os%20incentivos%20%C3%A0%20produ%C3%A7%C3%A3o	Product registration/marketing authorization route. The intended zootechnical or veterinary classification determines safety, quality, efficacy, and labeling requirements.
Authorization upon request	Preharvest	**Chile**<br>Certificate of Free Sale Servicio Agrícola y Ganadero (SAG)	NeoPhage-S® (Surphage)	Unknown regulatory identification code /Poultry production/Salmonella control	https://surphage.cl/	Free-sale certification/commercial evidence route: SAG certificate of free sale supports legal manufacture or commercialization/registration processes. It does not necessarily replace full health, efficacy, or marketing authorization in another jurisdiction.
Authorization upon request	Preharvest	**Chile**<br>Certificate of Free Sale Servicio Agrícola y Ganadero (SAG)	INSPEKTOR® PhageLab (Chile MSD Animal Health)	Unknown regulatory identification code Zootechnical solution / tailored phage cocktail + field support (poultry)	https://phage-lab.com/es/inspektor/<br>https://www.sag.gob.cl/sites/default/files/guia_para_solicitar_clv_23-03-2018.pdf#:~:text=El%20%E2%80%9Ccertificado%20de%20libre%20venta%E2%80%9D%20es%20un,regulares%20realizadas%20por%20el%20Servicio%20y%20es	Free-sale certification/commercial evidence route: SAG certificate of free sale supports legal manufacture or commercialization/registration processes. It does not necessarily replace full health, efficacy, or marketing authorization in another jurisdiction.
Authorization upon request	Preharvest	**Chile**<br>Certificate of Free Sale Servicio Agrícola y Ganadero (SAG)	FÓRMIDA® PhageLab (Chile MSD Animal Health)	Unknown regulatory identification code Zootechnical additive (poultry)	https://phage-lab.com/es/formida/<br>https://www.sag.gob.cl/sites/default/files/guia_para_solicitar_clv_23-03-2018.pdf#:~:text=El%20%E2%80%9Ccertificado%20de%20libre%20venta%E2%80%9D%20es%20un,regulares%20realizadas%20por%20el%20Servicio%20y%20es	Free-sale certification/commercial evidence route: SAG certificate of free sale supports legal manufacture or commercialization/registration processes. It does not necessarily replace full health, efficacy, or marketing authorization in another jurisdiction.

Note: Regulatory route clarification distinguishes between non-equivalent regulatory concepts, including product classification, health authorization, marketing or commercial authorization, free-sale certification, processing-aid/no-objection routes, and product registration. Product classification defines the legal category under which a bacteriophage-based product is assessed, while health authorization involves technical and sanitary evaluation. Marketing authorization allows commercialization after regulatory requirements are met, whereas free-sale certification usually confirms legal manufacture or commercialization in one country but does not necessarily replace safety or efficacy assessment in another jurisdiction. These categories should be interpreted according to the product's intended use, target matrix, claim, and application point along the farm-to-fork continuum.
